# Recent Advance in Biological Responsive Nanomaterials for Biosensing and Molecular Imaging Application

**DOI:** 10.3390/ijms23031923

**Published:** 2022-02-08

**Authors:** Zhenqi Jiang, Xiao Han, Chen Zhao, Shanshan Wang, Xiaoying Tang

**Affiliations:** 1 School of Life Science, Institute of Engineering Medicine, Beijing Institute of Technology, Beijing 100081, China; 7520200073@bit.edu.cn (Z.J.); katiehax@163.com (X.H.); zhaochen1011@126.com (C.Z.); 2 School of Chemistry and Chemical Engineering, Analysis & Testing Center, Beijing Institute of Technology, Beijing 100081, China; 00258103@bit.edu.cn

**Keywords:** biological responsive nanomaterials, biosensing, bioimaging, photoacoustic imaging

## Abstract

In recent decades, as a subclass of biomaterials, biologically sensitive nanoparticles have attracted increased scientific interest. Many of the demands for physiologically responsive nanomaterials in applications involving the human body cannot be met by conventional technologies. Due to the field’s importance, considerable effort has been expended, and biologically responsive nanomaterials have achieved remarkable success thus far. This review summarizes the recent advancements in biologically responsive nanomaterials and their applications in biosensing and molecular imaging. The nanomaterials change their structure or increase the chemical reaction ratio in response to specific bio-relevant stimuli (such as pH, redox potentials, enzyme kinds, and concentrations) in order to improve the signal for biologically responsive diagnosis. We use various case studies to illustrate the existing issues and provide a clear sense of direction in this area. Furthermore, the limitations and prospects of these nanomaterials for diagnosis are also discussed.

## 1. Introduction

Biological-responsive nanomaterials have higher bio-selectivity and bio-specificity than standard biomaterials, as well as added benefits of nontoxicity and non-invasiveness [1]. For the reasons stated above, biologically responsive nanomaterials have attracted a lot of interest in the disciplines of nanotechnology, innovative materials, medical imaging, and chemical sensing in recent decades [2,3,4,5]. These nanomaterials are revolutionizing the domains of nanoscience, material science, and even medicine [6,7]. Biological responsive nanomaterials are a class of ‘smart’ biomaterials that may modify their chemical structures or phases when exposed to environmental/external stimuli [8,9,10,11,12,13]. Biological responsive nanomaterials can respond to various bio-relevant stimuli (e.g., tissue-specific pH, redox potentials, and enzyme types and concentrations) as well as external stimuli (e.g., light exposure and heat). Upon the stimuli, biological responsive nanomaterials change their own structures or enhance the chemical reaction ratio in response to these stimuli, resulting in the change of the physicochemical properties of the materials (e.g., the surface charge, exposure of the cell-penetrating peptide or cell-targeting ligand, and control of drug release). As a result of these modifications, the physicochemical properties of nanomaterials are drastically altered. Biologically responsive nanomaterials can be thought of as minuscule “sense-to-action” devices capable of “reading in” and “reading out” input of external signals and output of information.

Drug delivery vehicles are one of the key applications of biological responsive nanomaterials [14]. They refer to micro/nano-carriers having specified shapes and dimensions, in general. Spherical micelles and hollow vesicles are two typical morphologies. By covalent or non-covalently encapsulating drug molecules within their interior, these nanoparticles can act as sophisticated cargo for achieving targeted/controlled medication transport and subsequent release. External signals can be easily adjusted to control the parameters (for example, locations, distributions, rates, and timing), allowing the disassembling of nanomaterials-based vehicles for “on-demand” medication delivery.

Many efforts have recently been directed towards studying biological responsive nanomaterials for highly sensitive biosensors and molecular imaging, with the increasing necessity for enhanced solution and sensitivity for clinical diagnostics [15]. For example, the Ye group designed biologically responsive magnetic resonance (MR) bimodal and near-infrared (NIR) fluorescence probes for molecular imaging by rational integration of a fluorogenic reaction within an enzyme-responsive in situ self assembly [16]. The underlined probe (P-CyFF-Gd) can be activated by overexpression of endogenous ALP on cellular membranes, resulting in assembled nanoparticles localized on membranes that can be observed by cryo-SEM. Due to simultaneous enhancements in r1 relaxivity (∼2.3-fold) And NIR fluorescence (>70-fold at 710 nm), highly sensitive, high-spatial-resolution imaging and localization of ALP activity within surviving tumor cells and mice is achievable. However, only a few studies have explored the use of physiologically sensitive nanomaterials in diagnosis.

The present study discusses the latest developments in biologically responsive nanoparticles for diagnostics (Table 1). We have primarily discussed the different kinds of biologically responsive nanomaterials and their prospective use in biosensing and molecular imaging for diagnostic purposes. This review excludes other bio-applications for biological responsiveness, such as medicine delivery, and wound healing.

## 2. Types of Biological Responsive Nanomaterials

Biologically responsive nanoparticles, such as polymer nanomaterials, inorganic nanomaterials, metal-organic frameworks, and carbon-based nanomaterials have emerged as a result of significant advancements in material science.

### 2.1. Organic Nanomaterials

Due to their intrinsic biocompatibility and biodegradability, organic nanoparticles have gained a lot of attention in the biomedical field [30]. Organic nanoparticles may detect and bind with targeted biological entities, such as proteins and peptides, with high affinity and selectivity in vitro and in vivo, making them a valuable biologically responsive nanomaterial [31]. Polymeric nanoparticles capable of selectively recognizing targeted proteins or cells can also be synthesized by immobilizing biologically responsive ligands (e.g., antibodies, peptides, nucleic acids, and small molecules) on the particles’ surface [32]. Several ligand-modified nanoparticles have been produced in recent years to influence cell-cell, protein-protein, and protein-cell interactions [33]. For example, Liu et al. experimented with an A recognition element-modified nanoparticle that was capable of altering the morphology of A aggregates, thereby leading to the development of co-assembled nanoclusters comprising A/nanoparticle, rather than A oligomers. This nanoparticle decreased A-induced neuron death by reducing pathogenic A oligomers [34]. Kim et al. produced a bio-specific multivalent nano-bioconjugate engager (denoted as mBiNE) using a substrate based on carboxylated polystyrene nanoparticles, which could potentially boost immune-mediated tumor cell eradication. mBiNE elicited robust and sustained anticancer T-cell responses against HER2+ malignancies and promoted HER2-targeted phagocytosis by concurrently immobilizing calreticulin (CRT, a phagocytic signal) and anti-HER2 antibodies onto the carboxylated polystyrene nanoparticles [33,35]. These studies showed polymeric nanoparticles have the capacity to mediate cell-cell, protein-cell, and protein-protein interactions.

### 2.2. Inorganic Nanomaterials

Inorganic nanomaterials are mainly comprised of inorganic components [36]. In comparison to organic or polymeric nanoparticles, inorganic nanomaterials have higher mechanical stability. Biologically sensitive inorganic nanoparticles (such as gold, silver, iron, platinum, titanium, cobalt, ceramic, and silica particles) have been in use for regenerative medicine applications in recent decades [37,38,39]. For example, in order to provide excellent biocompatibility and sufficient mechanical strength for bone tissue regeneration, a bone graft substitute should be capable of mimicking the ECMs of actual bone. Inorganic nanoparticles are a good candidate for bone graft replacement because of their high mechanical strength [40]. More importantly, inorganic nanoparticles can remain stable in the body for several weeks, assisting bone mending throughout the early stages of regeneration. Biologically responsive glasses, nanosilicates, hydroxyapatite, and silica nanoparticles have all been extensively used in bone tissue engineering. Nanohydroxyapatite, for example, has been shown to have a structure and chemical composition identical to bone tissue. As a result, nanohydroxyapatite allows the mesenchymal stem cells to detect it and adhere to it, making osteogenic differentiation easier [41]. Due to their antibacterial properties, metal-based nanoparticles have considerable potential in bone tissue regeneration regeneration [28,42]. Furthermore, Selvamurugan et al. reported a bio-composite scaffold for bone tissue engineering (referred to as the CS/nHAp/nCu-Zn scaffold) made of Cu-Zn alloy nanoparticles (nCu-Zn), nano-hydroxyapatite (nHAp), and chitosan (CS). They discovered that combining nano and micro layouts resulted in a suitable surface for bone tissue development and cell penetration. The supplementation of nCu-Zn to the CS/nHAp scaffold led to greater swelling, decreased degradation, and higher antibacterial activity when compared to the CS/nHAp sacaffold [42,43]. However, toxicity, which is mostly caused by long-term non-specific accumulation of inorganic nanomaterials in organs and normal tissues, is a barrier to their usage in therapeutic applications.

### 2.3. Carbon-Based Nanomaterials

Carbon-based nanomaterials have attracted attention in recent decades, with biomedical applications being investigated. Carbon nanotubes, graphene, and graphene oxide, among these nanomaterials, have gotten a lot of attention because of their unusual structural and mechanical characteristics [44]. Carbon nanotubes and graphene are now commonly used in tissue engineering [45]. Carbon nanotubes are carbon atoms-based molecular-scale hollow tubes that offer a great degree of mechanical strength and flexibility. Tan et al. revealed that carboxylated multi-walled carbon nanotubes (MWCNTs) may improve neurogenic differentiation and cell adhesion in mesenchymal stem cells without inducing factors, but had no effect on osteogenic differentiation [46,47]. Watari et al., on the other hand, found that when differentiation-inducing factors are not present, MWCNT films could efficiently stimulate mesenchymal stem cells’ osteogenic differentiation [47]. Carbon atoms are organized in a honeycomb lattice in a one-atom-thick membrane referred to as graphene. Graphene, in contrast with carbon nanotubes, has a large specific surface area and an open surface, making it ideal for non-covalent interactions with biomolecules or chemical ligand modification. In comparison to conventional materials, graphene has a larger Young’s modulus (E, 0.5–1 TPa), although it is not brittle [48]. Hence, graphene stands out as a superior material for bone and tissue regeneration. A highly oxidized form of graphene, graphene oxide, is made by oxidizing graphite, and it’s been used in regenerative medicine and other biomedical applications [49]. Bai et al., for example, demonstrated that polydopamine-functionalized reduced graphene oxide can promote biomimetic hydroxyapatite mineralization (denoted as RGO-PDA). In MC3T3-E1 cells on RGO-PDA substrates, osteogenic differentiation, cell adhesion, and proliferation were all higher in comparison to that of bare [50]. Yang et al. demonstrated that graphene oxide may successfully induce autophagy in microglial cells and neurons in vitro and in vivo by inhibiting the mTOR signalling pathway [51]. Although carbon compounds have great importance in literature and medicine and are thought to be inert to cells and tissues, their reactivity increases dramatically at the nanoscale. Hence, future studies will need to investigate the possible toxicity of carbon-based nanomaterials.

### 2.4. Metal-Organic Frameworks

As porous materials comprising organic ligands and metal ions or clusters, metal-organic frameworks (MOFs) involve self-assembly through coordination bonding. MOF materials are available in a wide range of shapes and sizes, with high porosity, tunable pores, and specific surface area, and can be used in various applications, including energy storage and catalysis [52,53]. In contrast to typical porous materials, nanoscale metal-organic frameworks (NMOFs) have huge surface areas, adjustable pore sizes, and well-defined crystalline structures [54,55,56,57]. The controlled synthesis and potential use of NMOFs have been widely investigated in the synthesis of MOFs so far. Post-synthetic stirring of the drug with the dried ZIF-8 powder in aqueous solution enabled the junior group to effectively load ZIF-8 with DOX (4.9 wt %) [58]. Release of the drug occurred in a progressive and highly regulated manner (66% drug release after 30 days). Similarly, ZIF-8 was employed as a pH-responsive drug carrier for 5-fluorouracil (5-FU) [59]. The drug’s extraordinary potency was produced by post-synthetically modifying ZIF-8 with 5-FU, which yielded roughly 660 mg of 5-FU/g of ZIF-8. The studies revealed that the release of drug was faster in a mildly acidic buffer (pH = 5.0) than in a neutral buffer solution (pH = 7.4). We described the production of a pH-responsive and targeted nano-carrier by conjugating the Y_1_ receptor ligand [Asn6, Pro34]-NPY (AP) on the surface of ZIF-90 through a Mannich reaction, followed by loading DOX into ZIF-90 pores. The carrier detected and treated triple-negative breast cancer cells specifically [60]. The drug loading capacity was 12.6%, estimated by one-pot encapsulation in the DOX loading experiment. The coordination link between Zn^2+^ and adenosine triphosphate (ATP) is substantially stronger in comparison to that between Zn^2+^ and imidazole, and AP-ZIF-90-DOX exhibited dual pH and ATP-responsive drug release. At pH 7.4, the release of DOX occurred only up to a magnitude of 1.7% from AP-ZIF-90@DOX, whereas with the addition of 0.5 mM ATP, 19.8% DOX was released, as depicted by the in vitro release experiments. Following 2 h at pH 5.0 with 0.5 mM ATP, more than 21.7% DOX was released, causing the ZIF-90 to collapse under the acidic situation. AP-ZIF-90@DOX demonstrated good biocompatibility in the mouse model during 30 days of in vitro and in vivo cytotoxicity testing. Further investigation of the in vivo metabolic pathway and metabolic mechanism of MOFs, as well as extensive investigation and long-term monitoring of their biological safety, is required.

## 3. Biological Responsive Nanomaterials for Biosensing

The conversion of biological or chemical entities into quantifiable signals is referred to as bio-sensing. The biological responsive nanomaterials are projected to boost the effect of biosensing, including both enzyme-based and enzymeless-based biosensing, due to their higher biological responsiveness and post-synthesis capabilities.

### 3.1. Enzyme-Based Biosensing

**Figure 1:**
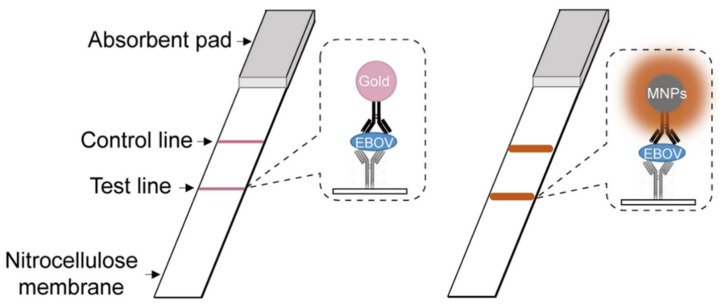
Schematic illustration of Fe_3_O_4_ nanozyme-strip for the detection of EBOV. Adapted from ref [18], with permission from Copyright © 2015, Elsevier B.V. All rights reserved.

The most widely used technique in biosensing is colorimetry, which uses peroxidase as a transducer to catalyze the oxidation of colorless peroxidase substrates into colored entities [61]. The first report of the intrinsic peroxidase-mimicking capabilities of Fe_3_O_4_ magnetic nanoparticles (MNPs) was given by the Yan group in 2007 [62], while in the following 10 years, dozens of carbon, noble metal, vanadium, and MOF-based nanozymes have been shown to manifest similar mimicry characteristics [63]. When compared to natural enzymes, these nanozymes had higher catalytic activity, lower costs, and better physical/chemical stability, indicating that nanozymes have a lot of potential in biosensing. By interacting with multiple receptors, nanozymes may detect a variety of chemical and biological species. Nanozymes, for example, can successfully detect their substrates when paired with oxidases. By using glucose oxidase (GO_x_) as the receptor, Wei et al. presented a ZIF-based nanozyme GO_x_/hemin@ZIF-8 capable of detecting glucose in beverages, urine, and blood [19]. Xu et al. described a copper sulfide-based nanozyme BNNS@CuS capable of detecting human serum levels of total cholesterol visually by using cholesterol oxidase as a receptor [64]. Nanozymes can also detect antigens that have been specifically targeted. In a seminal paper, Yan’s group combined the two properties of Fe_3_O_4_ nanozyme, peroxidase and magnetism, and reported a unique capture–detection immunoassay (Figure 1) [18,62]. This antibody-conjugated Fe_3_O_4_ nanozyme can undergo binding with antigen in mixes, then a magnet field was used to remove the antigen from the specimen, resulting in a colorimetric signal. This method has captured, separated, and detected TnI and EBOV by using an anti-EBOV antibody and an anti-cardiac troponin I (TnI) antibody as receptors. They recently designed a unique Co–Fe@hemin nanozyme and loaded anti-SARSCOV-2 antibody into a chemiluminescence immunoassay of SARS-CoV-2 antigen in serum. By making use of specific cell markers as targeted antigens, nanozymes can also detect a specific cell phenotype. Gao et al. developed a nanozyme-based probe to measure integrin GPIIb/IIIa expression levels on cell surface [65]. This peptide-conjugated AuNP (H_2_N-CCYKKKKQAGDV-COOH) can attach to integrin, thereby generating a colorimetric signal. As a result, the level of integrin expression on human erythroleukemia cells may be measured using a colorimetric method that requires no protein extraction or cell lysis. It has been reported by Chen et al. that platinum nanoparticles and graphene oxide (PtNPs/GO) may be utilized to identify cancer cells [17]. By accelerating TMB oxidation in the presence of H_2_O_2_, the folic acid (FA) on PtNPs/GO can preferentially undergo binding with FA receptors present on cell membranes and provide a colorimetric signal in situ. Other distinctive indicators, such as glycans and epithelial cell adhesion molecule (EpCAM) in addition to protein receptors, can be utilized for cell detection by combining with their biological recognition. Lectin and Anti-EpCAM aptamer (SYL3C) are examples of ligands [66,67].

### 3.2. Enzymeless-Based Biosensing

Enzyme-free sensors are less influenced by environmental conditions and can be stored for longer periods of time than enzyme-based sensors [68]. The development of new electronic mediators and nanomaterials, as well as their use in the development of electrochemical biosensors, has sparked interest in this field. Carbon nanoparticles are amongst the most widely employed materials and the most typical of nanomaterials utilized in the development of non-enzyme-based electrochemical sensors, amongst the multitude of biomaterials utilized for the purpose [69]. Metal-organic frameworks, Carbon nanomaterials, and metal nanoparticles have all been used as non-enzyme biosensors in recent years [70,71].

**Figure 2:**
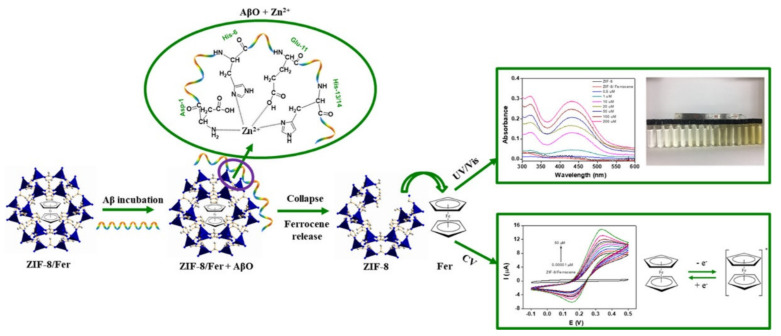
Nanoscale ZIF-8/Fer for AβO sensing utilizing electrochemical and optical methods. Adapted from ref [21], with permission from Copyright © 2019, American Chemical Society.

Curcumin’s hydrophobic groups have been shown in reported studies to strongly engage with the non-polar portions of Aβ oligomers by means of hydrophobic contacts. As a result, curcumin is an excellent candidate for identifying Aβ [72]. Qin et al. developed two curcumin and curcumin Ni-based non-enzyme electrochemical AβO sensors [22]. For the first time, they used a curcumin-based biosensor to detect AβO. Using these sensors, curcumin is polymerized electrochemically on nickel foam. Cyclic voltammetry (CV) method was employed to electrochemically polymerize the curcumin/curcumin-Ni complex on Ni foam, with the number of cycles regulating the polymer formation. Curcumin inhibits amyloid aggregation due to the hydrophobic interaction of the aromatic residues in the Aβ peptide with curcumin and the presence of many intermolecular hydrogen bonds. Using electrochemical impedance spectroscopy (EIS), the sensitivity of AβO detection for the polycurcumin electrode was determined to be in the 0.01–1 nM range, the limit of detection (LOD) being 0.01 nM. Poly (curcumin-Ni) electrode had a high sensitivity, and AβO detection sensitivity was in the range of 0.001–5 nM; LOD being equivalent to 0.001 nM determined using EIS. Lower AβO concentrations can be detected using this stage of curcumin electropolymerization. As a result, it could be a potential technique for detecting Alzheimer’s disease in the early stage. Qin and his colleagues also attempted the pioneer investigation on ZIF/Fer to see if it could be used as an AβO non-enzyme electrochemical sensor [21]. Ferrocene-encapsulated ZIF-8 (ZIF-8/Fer) was developed for dual detection using electrochemical and optical techniques by self-assembly of MIM and Zn ions in the presence of ferrocene (Figure 2). The coordination of several amino acids such as glutamic acid (Glu), histidine (His), and aspartic acid (Asp) with Zn^2+^ in Aβ_1−42_ made the Zn-ZIFs sensitive to Aβ_1−42_, and ferrocene was released in a concentration, linearly proportional to the AβO content, as ascertained by CV and UV/vis spectroscopy. The qualitative monitoring of AO was accomplished by molecular optical sensing, whereas the quantitative detection was accomplished through electrochemical analysis. In the quantitative as well as qualitative determination of AβO, the use of dual detection in conjunction with the two sensing technologies has a synergistic impact. The ferrocene-encapsulated ZIF-8/Fer was shown to have a low LOD and might aid in detecting AβO over an extensive range. The detection range of Aβwas determined to be 0.5–100 mM with a LOD of 0.5 mM using UV/Vis spectroscopy. However, making use of an electrochemical approach, the LOD was improved to 10–5 mM and was appropriate in the region of 10^−5^–10 mM. (Figure 2).

As a bifunctional brain biomolecule, Serotonin (5-hydroxytryptamine, 5-HT) acts as both a neurotransmitter and a hormone [73]. Any impairment in 5-HT signaling is linked to anxiety and depression, and also has a significant contribution in the pathogenesis of several age-related diseases, such as AD and HD and insulin resistance [74]. However, 5-HT coexists with a variety of biological chemicals, including as dopamine, electrochemical sensors that can detect 5-HT selectively are necessary. Al-Graiti et al. reported a form of ribbon sensor capable of simultaneously detecting dopamine and 5-HT dopamine, that comprised a conductive polymer layer and nanostructured hybrid graphene [75]. The rGO–PEDOT/PSS sensor can detect 5-HT over a concentration range of 0.1–10 mM, having a LOD of 0.1 mM utilizing CV. Furthermore, dopamine has little effect on analysis.

Glucose is one of the most abundant monosaccharides on the planet. As the biological centre of glucose enzyme sensors, oxidoreductase enzymes are split into two categories: glucose dehydrogenases (GDHs) and glucose oxidase (GO_x_). GDHs can be subsequently categorized based on their redox cofactors [76]. In contrast, enzyme-based sensors have a number of disadvantages. Urea and other biomolecules may be oxidized in addition to glucose due to the high voltage necessary for this test. The effects of dissolved oxygen or oxygen partial pressure on the experimental results are significant, and the high concentration of hydrogen peroxide produced by the reaction causes the enzyme activity to diminish or inactivate. To detect glucose, Lu et al. utilized a commercial Ni foam which functioned as the working electrode. The sensor had a 0.05–7.35 mM linear range, with a 2.2 mM detection limit, making it the most basic Ni-based electrochemical glucose sensor [77]. Sun et al. successfully synthesized Co_3_N and CoP and then improved the sensor’s performance by using the superior conductive qualities of transition metal nitride and phosphide [78]. The detection limits for Co_3_N and CoP based glucose sensors were 3325.6 mA mM^−1^ cm^−2^ and 5168.6 mA mM^−1^ cm^−2^, respectively, while the sensitivity was 3325.6 mA mM^−1^ cm^−2^, respectively [79]. Ye et al. constructed the sensor using Cu cubes wrapped in a carbon shell [80]. The sensor’s linear range is 40 mM to 40 mM, the sensitivity is equivalent to 2565 mA mM^−1^ cm^−2^ whereas its detection limit is 21.35 mM.

## 4. Biological Responsive Nanomaterials for Molecular Imaging

TME-responsive DINAs have been extensively employed in bioimaging, including photoacoustic imaging, fluorescence imaging, and MRI, due to their capacity to lengthen blood circulation duration, optimize distribution within the body, and intelligently switch functions as well as structures. 

### 4.1. MR Imaging

**Figure 3:**
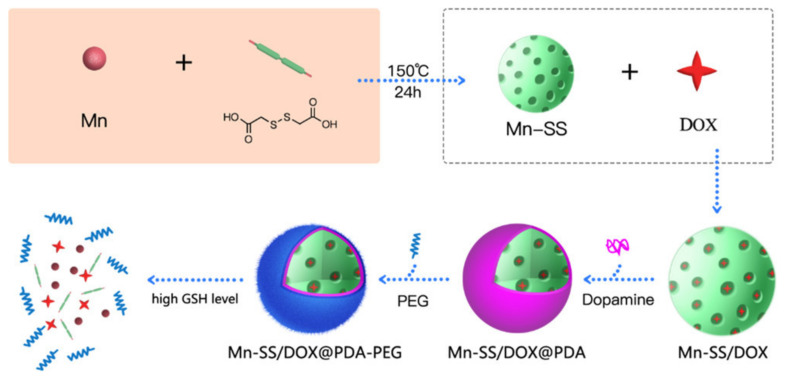
Synthesis and characterization of Mn-SS NCPs, as well as the GSH, triggered nanoparticle decomposition, drug release, and Mn^2+^-enhanced MRI. Adapted from ref [27], with permission from Copyright © 2017, American Chemical Society.

MRI has been widely employed in clinical diagnostics as a general imaging [81]. To improve the imaging contrast of MRI, magnetic contrast agents, such as iron oxide nanoparticles and gadolinium (Gd)-containing complexes, have been used [82,83,84]. The employment of biologically responsive magnetic nano-assemblies with stimuli-responsive image contrasting characteristics to improve cancer imaging has been extensively used. T_1_-weighted MRI in acidic solid tumors can be improved by utilizing pH-responsive iron oxide nanoparticle assemblies [85]. Furthermore, MRI signals can be improved by in-situ production of magnetic nano-assemblies in tumor tissues. For example, Sun’s research group, established a grafting/peeling-off approach in which MMP-2 cleavable fluorescent dye-PEG ligands were used to modify core/shell iron/iron oxide nanoparticles (Fe/IONPs) for better tumor-targeted MRI imaging [86]. The Fe/IONPs could, for starters, passively accumulate at tumor areas due to the EPR effect. Detachment of the PEG from the surface of Fe/IONPs occurred due to MMP-2 enzyme-responsive colloidal behaviors, resulting in agglomeration of Fe/IONPs into substantially-sized assemblies within the tumor, permitting long-term tumor retention. As a result, these Fe/IONPs in-situ assemblies have been used for non-invasive monitoring and detection of MMP-2 overexpressed tumors using MRI. In tumor MRI, biological responsive bioimaging has also produced positive outcomes. Gao and colleagues reported in-situ crosslinking of Fe_3_O_4_ nanoprobes to create glutathione (GSH)-responsive nano assemblies for enhanced tumor MR imaging [87]. To begin, the surface of Fe_3_O_4_ nanoparticles was modified with self-peptide and disulfide-linked RGD peptides. The self-peptide at the top layer, which has stealth coating qualities, could limit nanoparticle clearance in the bloodstream. As GSH levels rise in the tumor microenvironment, the disulfide bond may be disrupted, exposing active groups to crosslink the Fe_3_O_4_ particles in situ. The MRI contrast enhancement performance for in vivo tumor identification may be accomplished after the crosslinking of Fe_3_O_4_ particles in the tumor. For drug delivery and cancer theranostics, redox-sensitive nanoscale coordination polymers (Mn-SS NCPs) were synthesized using Mn^2+^ and S-S-containing organic bridging ligands [27]. GSH cleaves the S-S bond, allowing for effective redox-triggered drug release and robust T1-weighting MR imaging (Figure 3).

### 4.2. Luminescence Imaging

The advantages of luminescence imaging include low radiation, low invasiveness, real-time rapid reaction, low toxicity, and excellent spatial imaging potential. In the construction of GSH-responsive prodrug probes, Ye et al. employed a fluorophore based on a cyanine dye (em = 650 nm), which successfully enhanced the fluorescence wavelength and enabled effective in vivo imaging [88]. Tan et al. developed the first single-molecule prodrug probe (PNPS), which combined photodynamic therapy and chemotherapy in a single-molecule system [89]. A H_2_O_2_-responsive group bis-borate was used as a bridge to connect the anticancer medication 50-deoxy-5-fluorouridine and an NIR photosensitizer (NPS) in the probe PNPS (50-DFUR). Low fluorescence intensity and toxicity, as well as excellent mitochondrial targeting, were also the key features of the system. With a higher concentration of H_2_O_2_, PNPS can efficiently undergo activation within cancer cells, and the controlled release of chemotherapeutic medications and NIR fluorescent groups subsequently follows, in order to achieve the effects of photodynamic treatment and chemotherapy at the tumor location. The probe PNPS shows good tumor microenvironmental response, as suggested by in vivo fluorescence imaging and cell viability tests.

Photon-activated prodrug probes function by the activation and regulation of endogenous chemicals. This results in a fluorescent prodrug probe that is light-controlled. The light-excited prodrug probe works on the following principle: the prodrug is first injected into the circulating body fluid to build up to a pre-determined concentration within the lesion site. Local laser irradiation is then used to begin activation of the prodrug, causing it to change fluorescent signals ad release chemotherapeutic drugs. A light-activated probe can efficiently decrease the harm to natural tissue while also achieving the goal of precision treatment [90]. Cy-CPT-biotin, the first prodrug probe activated by near-infrared light, was synthesized by Zhu et al. using basic organic chemistry procedures [91]. Fluorescence emission at 810 nm could be utilized to track the distribution of the prodrug. The active drug CPT is released by the residual structure and creates Cy-biotin with a fresh emission at 535 nm via continuous intramolecular cyclization when the polyolefin bond breaks due to external light irradiation at the tumor site. Cy-CPT-Biotin demonstrated superior tumor targeting and photo-controllable cytotoxicity in both in vivo and in vitro tests, successfully decreasing systemic toxicity. Liu et al. created a fluorescent prodrug probe that is activated by two photons [92]. It was definitely an improved version since it was capable of extending the incident laser to a wavelength of 800 nm, thereby resulting in an effective decrease in the phototoxicity resulting from shortwave radiation.

### 4.3. Photoacoustic Imaging

Photoacoustic imaging (PA) is a noninvasive in vivo imaging technique that delivers superior resolution and sensitivity, allowing for deeper tissue penetration than traditional optical imaging [93,94]. The development of biologically responsive PA imaging nanoprobes will aid cancer diagnosis and tracking of therapy response. The Liu group developed the MnMoO_x_ bimetallic oxide in an attempt to precisely detect glutathione (GSH) in solid tumors [95]. The MnMoO_x_ can barely absorb near-infrared (NIR) light without GSH. While the Mo(VI) in the MnMoO_x_ could be reduced to Mo (V) in the TME in the presence of GSH at a high concentration, the MnMoO_x_ would be broken down to ultrasmall nanodots with outstanding NIR absorbance for PA imaging. It is noteworthy that the MnMoO_x_ might accomplish fast renal clearance due to its inherent biodegradability. Similarly, molybdenum-based polyoxometalate clusters with the highest oxidation state of Mo (VI) (Ox-POM) were developed for redox-activated PA imaging [96]. Since in the tumor redox milieu, Mo (VI) is reduced to Mo (V), the Ox-POMs would presumably have substantial NIR absorption for PA imaging, similar to the MnMoO_x_. In another scenario, MnO_2_-based nano assemblies that are capable of absorbing a broad spectrum of light can be used to act as an efficient contrast reagent for PA imaging [97]. When cancer cells are exposed to high levels of GSH, the black MnO_2_ nano assemblies are converted to colorless Mn^2+^ ions, lowering the PA amplitude and enabling dynamic monitoring of GSH levels.

**Figure 4:**
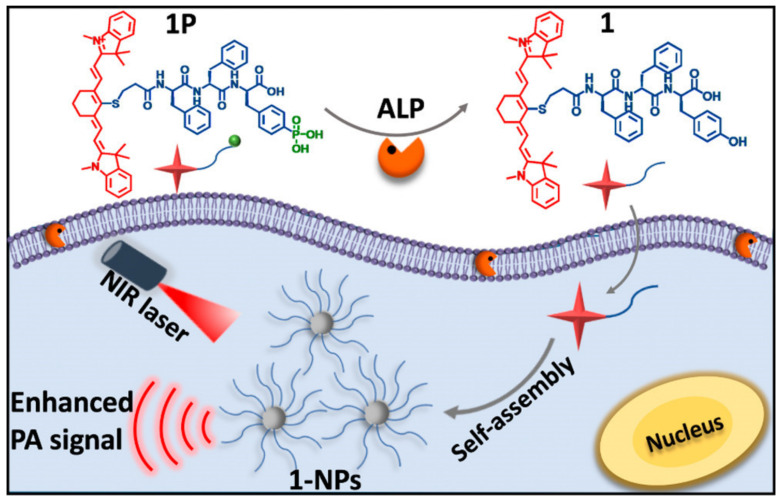
ALP-triggered self-assembly of near-infrared nanoparticles for the enhanced PA imaging of tumors. Adapted from ref [29], with permission from Copyright © 2018, American Chemical Society.

Meanwhile, a pH-responsive albumin-based nanoprobe was developed by Liu et al. for ratiometric photoacoustic pH imaging in vivo [98]. pH-responsive probes were generated by co-loading IR 825 dyes and benzo[a]phenoxazine (BPO_x_) on self-assembled human serum albumin (HSA). Due to its protonation-enhanced absorption, BPOx functions as a pH indicator in this nanosystem, and the IR 825 dye serves as an internal reference for ratiometric PA imaging. According to in vivo PA imaging, the tumor had a significantly higher ratio of PA680 to PA825 signals when compared to normal tissue. Furthermore, Wu et al. developed the IR775-Phe-Phe-Tyr(H_2_PO_3_)-OH (1P) NIR probe for PA imaging ALP activity in vitro and in tumor [29]. Due to ALP’s catalysis, 1P was successfully transformed to IR775-Phe-Phe-Tyr-OH (1), which self-assembled into the nanoparticles **1-NPs**. The production of 1-NPs resulted in a 6.4-fold increase in the PA signal of **1P** at 795 nm. According to in vivo tumor PA imaging studies, PA contrast in the experimental group increased 2.3 times at 4 h following **1P** injection compared to the ALP inhibitor-treated control group (Figure 4).

### 4.4. Other Types of Imaging Modalities

Surface-enhanced resonance imaging is another biologically responsive bioimaging technique. Additionally, computed tomography (CT) and Raman spectroscopy (SERRS) have been employed for tumor diagnosis and treatment [99,100]. A pair of gold nanospheres (Au-AK and Au-AZ) were used to guide brain-tumor surgery by simultaneous activation of both SERRS and MR signals following in-situ construction when exposed to the acidic tumor [101]. At first, Au–AZ and Au–AK nanoprobes were monodispersed within the circulating blood, and both of them have the potential to penetrate brain tumors by surpassing the blood-brain barrier (BBB) via receptor-mediated transcytosis (RMT) mediated by lipoprotein-receptor related protein-1 (LRP1). Within the acidic TME, the shielding layer of the nanoprobes would be dissolved, exposing alkyne and azide groups on their surface, allowing the nanoparticles to combine and activate SERRS and MR signals. Intraoperative and preoperative dual-modal imaging with pH-responsive nanoprobes could potentially be utilized to guide brain tumor removal.

CT contrast agents based on heavy metallic nanoparticles, for instance, gold, bismuth, and hafnium have been created for tumor-targeted CT imaging [102]. The EPR effect has recently been used to produce tumor-targeted CT imaging using elongated BNTs nano assemblies with high aspect ratios made up of ultrasmall bismuth subcarbonate nanoclusters (BNCs) [103]. Following that, these BNTs were disassembled into separate nanoclusters in response to the acidic TME, hence increasing renal excretion. As a result, the renal clearable CT contrast agent having quick clearance characteristic showed a considerable role in biomedical applications.

## 5. Conclusions and Future Perspectives

Biological responsive nanomaterials are a type of nanoscale biomaterial that has the potential to trigger a biological reaction when interacting with proteins, tissues, and cells. The bioactivities of biological sensitive nanomaterials are influenced by a variety of factors, including material physical structure and surface quality. These characteristics have a substantial impact upon the interactions of biological systems with nanomaterials, resulting in a variety of biological responses. Due to their distinct bioactivities, multiple biological responsive nanomaterials have emerged for tissue regeneration and for treating various diseases over the last decade. Biological responsive nanomaterials and their tunable nanostructural frameworks enable a wide range of bioactivities and biomedical applications due to their unique physicochemical properties. To date, biologically sensitive nanomaterials have been fully investigated for a variety of bio-sensing and bio-imaging applications. However, considerable barriers to the production and widespread use of physiologically sensitive nanomaterials remain, and more research in the areas indicated below is required.

(1) Additional chemical mechanism research: present efforts are mostly focused on the production of appealing biologically sensitive nanomaterials and the investigation of their possible uses. Chemical mechanisms, on the other hand, have gotten less attention in material design, especially in the case of carbon-based nanomaterials and inorganic nanomaterials. More research related to the chemical pathways could aid researchers in gaining an improved understanding of the structure-activity relationship, allowing for the intelligent design and production of optimum biologically responsive nanomaterials.
(2) Expanding the breadth of biologically responsive nanomaterials: Many studies focus on the bioactivities of classic nanomaterials, which have been discussed in this review. An increasing number of biomaterials with precise nanostructures, especially DNA-based materials, have been developed in recent years. Future research should focus on the new biomaterials’ physiochemical properties and bioactivities. The nanoparticles created using 3D printing technology have the potential to be important biologically sensitive nanomaterials, and their bioactivities should be assessed. Natural nanomaterials’ bioactivities should also be investigated owing to their outstanding biocompatibility and readily available sources.
(3) Non-enzyme electrochemical biosensors: non-enzyme electrochemical biosensors’ specificity and sensitivity must be improved due to the lack of enzymes within their design; hence, there is a critical need to identify materials for the development of sensors that can detect biomarkers even in the presence of interfering molecules.
(4) Clinical translational research: clinical applications that necessitate the collaboration of diverse experts from material sciences, medical sciences, life sciences, and pharmacy are rarely investigated. For example, the acute and chronic toxicity of biologically sensitive nanomaterials should be investigated further. Their scale-up preparation, sterilization, and storage, all of which are critical for clinical practice, must be prioritized.

## Figures and Tables

**Table 1:** Advanced biological responsive nanomaterials and their diagnostic application.

Type of Diagnosis	Materials	Types	Bio-Relevant Stimuli	Application	Ref.
Biosensing	Peptide conjugated Au NP	Inorganic nanomaterials	Protein	Immunoassays	[17]
	Fe_3_O_4_ MNP	Inorganic nanomaterials	H_2_O_2_	Immunoassays	[18]
	GO_x_/hemin@ZIF-8	Metal-organic Frameworks	Glucose	Biosensing	[19]
	Platinum NPs/graphene oxide	Carbon-based nanomaterials	Protein	Cancer cell detection	[20]
	ZIF-8-ferrocene	Metal-organic Frameworks	AβO	Electrochemical sensing	[21]
	Polycurcumin	Organic nanomaterials	AβO	Electrochemical sensing	[22]
	rGO–Cu_2_O/GCE	Carbon-based nanomaterials	dopamine	Electrochemical sensing	[23]
	Pt/PANI/rGO/CuO	Carbon-based nanomaterials	Glucose	Electrochemical sensing	[24]
	PdCu alloy	Inorganic nanomaterials	Glucose	Electrochemical sensing	[25]
Molecular imaging	MnO_2_ nanoplatforms	Inorganic nanomaterials	pH	MRI	[26]
	Mn-SS/DOX@PDA-PEG	Organic nanomaterials	Glutathione	MRI	[27]
	Albumin-Based Nanoprobe	Organic nanomaterials	pH	Photoacoustic imaging	[28]
	IR775-Phe-Phe-Tyr(H_2_PO_3_)-OH	Organic nanomaterials	alkaline phosphatase	Photoacoustic imaging	[29]
